# Changes in Comorbid Conditions After Prolonged Exposure for PTSD: a Literature Review

**DOI:** 10.1007/s11920-015-0549-1

**Published:** 2015-03-05

**Authors:** Agnes van Minnen, Lori A. Zoellner, Melanie S. Harned, Katherine Mills

**Affiliations:** 1 Behavioural Science Institute, Radboud University Nijmegen, NijCare, Pro Persona, Tarweweg 2, 6524 AM Nijmegen, The Netherlands; 2 Department of Psychology, University of Washington, Seattle, WA USA; 4 National Drug and Alcohol Research Centre, University of New South Wales, Sydney, Australia

**Keywords:** PTSD, Trauma, Exposure therapy, Comorbidity, Secondary outcomes, Worsening

## Abstract

Prolonged exposure (PE) is an effective psychological treatment for patients who suffer from PTSD. The majority of PTSD patients have comorbid psychiatric disorders, and some clinicians are hesitant to use PE with comorbid patients because they believe that comorbid conditions may worsen during PE. In this article, we reviewed the evidence for this question: what are the effects of PE on comorbid symptoms and associated symptomatic features? We reviewed findings from 18 randomized controlled trials of PE that assessed the most common comorbid conditions (major depression, anxiety disorders, substance use disorders, personality disorders, and psychotic disorders) and additional symptomatic features (suicidality, dissociation, negative cognitions, negative emotions, and general health and work/social functioning). Although systematic research is not available for all comorbid populations, the existing research indicates that comorbid disorders and additional symptomatic features either decline along with the PTSD symptoms or do not change as a result of PE. Therefore, among the populations that have been studied to date, there is no empirical basis for excluding PTSD patients from PE due to fear of increases in comorbid conditions or additional symptomatic features. Limitations of the existing research and recommendations for future research are also discussed.

## Introduction

Comorbidity is high in PTSD patients, as 80–90 % of individuals with PTSD have one or more comorbid conditions and two thirds have two or more additional diagnoses [1]. The most common comorbid diagnoses include major depression, anxiety disorders, substance use disorders, borderline personality disorder, and psychotic disorders. In addition, some patients show additional symptomatic features such as suicidality, dissociation, distorted and negative trauma-related cognitions (e.g., self-blame, perceptions of the world as extremely dangerous), persistent negative trauma-related emotions (e.g., anger, guilt, shame), physical health problems, and limited work and social functioning. Given that comorbidity is the norm rather than the exception among PTSD patients, an important question to answer is how to optimally treat PTSD patients with these comorbidities and associated features.

Prolonged exposure (PE) is recommended as a first-line treatment approach for PTSD (e.g., International Society for Traumatic Stress Studies [2]; National Institute for Health and Clinical Excellence Guidelines on PTSD [3]; for a meta-analysis of PE, see [4]). The primary components of PE include imaginal exposure to the trauma memory followed by processing of the trauma memory as well as in vivo exposure to feared but safe situations. The theorized mechanisms underlying PE are based on emotional processing theory and broader extinction models of fear reduction. Specifically, dysfunctional meaning associations underlying the trauma-related fear are altered or disconfirmed via information obtained during the exposure exercises [5].

PE is a trauma-focused approach, which means that the focus is primarily on processing the memory of the trauma and its effects on the patient’s life and, accordingly, to directly target trauma-related symptoms such as PTSD. Given this focus, comorbid disorders or problems are not (or minimally) addressed before or during treatment. Despite its efficacy, PE is underused by clinicians, especially in cases of comorbidity [6, 7]. One of the reasons clinicians may be hesitant to use PE is due to fear that comorbid conditions, when left untreated, will interfere with trauma-focused treatment. Another reason for not using trauma-focused treatment approaches such as PE is that clinicians are afraid that the comorbid conditions will worsen when the trauma is directly processed.

In a previous review [8••], we addressed the first concern of clinicians, namely, whether comorbid conditions contraindicate the use of PE. The conclusion was that, although more controlled studies are necessary, it is possible to effectively and safely treat PTSD patients with many comorbid conditions (e.g., major depression, dissociation, substance abuse) with PE and without directly treating the comorbidity itself. For more severe comorbidities such as serious self-injurious behavior, acute suicidality, and substance dependence, it was concluded that integrated or concurrent treatments that include PE in combination with treatments or strategies targeting the comorbid problem might be optimal. However, this prior review did not address the second concern raised by clinicians, namely, does PE lead to worsening of comorbid disorders and associated problems? In the present review, we will address the question of how comorbid symptoms and commonly associated features change as a result of PE.

There are several models to explain the relationship between PTSD and high comorbidity rates. It should be noted that comorbidity technically refers to two separate underlying causal entities existing simultaneously but independently [9]. However, for the purposes of this paper, we use the term comorbidity to imply a shared association between two constructs. Some causal models propose that PTSD mediates the relationship between trauma exposure and other comorbid problems. For example, PTSD has been hypothesized to mediate the effects between traumatization and the course and severity of severe mental illness both directly (through specific PTSD symptoms) or indirectly (through correlates of PTSD [10]). Similarly, PTSD symptoms of re-experiencing and avoidance/numbing have been found to mediate the relationship between childhood sexual abuse and non-suicidal self-injury [11]. Other theoretical models state that PTSD and comorbid conditions can be accounted for by common underlying etiologies or vulnerabilities (see for an overview [12•]). For example, childhood abuse has been implicated in the development of both PTSD and borderline personality disorder (e.g., [13, 14]) and depression and PTSD have been found to share an underlying factor of negative affect (see e.g., [15]). Also, reciprocal models have been proposed, suggesting that PTSD may influence comorbid conditions and vice versa. For instance, substance abuse may lead to high-risk behavior, which can lead to victimization and subsequently PTSD. Conversely, PTSD may lead to higher substance use as a means of self-medicating (see also [16]). In addition, explanations for the high comorbidity could be more conceptual and due, for example, to symptom overlap. This has been argued in the case of PTSD and comorbid major depression, as the two disorders share common symptoms such as anhedonia, insomnia, and difficulty concentrating (e.g., [17]). Recently, a network model of comorbidity was explicated by Borsboom and colleagues [18] that proposed that comorbidity arises due to causal relations between symptoms. The pathways between these symptoms may be different for each person, which helps explain the complexity in studying comorbidity, although general patterns may arise from the network analyses. For PTSD, for instance, Frewen and colleagues [19] found evidence for a perceived causal role of the re-experiencing of traumatic memories in exacerbating symptoms of depression. The complexity of studying comorbidity in PTSD is further illustrated by the fact that the number of symptom combinations (PTSD symptoms in combination with symptoms of comorbidity) as currently defined by the DSM-5 is over one quintillion [20].

In conclusion, comorbidity between PTSD and other mental disorders is high, the relationships between PTSD and other comorbid conditions is complex, and much more research is needed to unravel possible underlying mechanisms. Importantly, however, regardless of the specific theoretical model explaining the relationship between PTSD and comorbidity, in our view, current models do not predict that comorbid symptoms will *increase* as a result of trauma-focused treatment, as many clinicians may fear. On the contrary, one of the clinical implications of these theoretical models about comorbidity is that, when the PTSD symptoms are successfully treated, comorbid symptoms will decrease or, at least, stay stable.

## Review Methods

In this article, we will review the secondary outcomes of studies that target PTSD symptoms using PE. Therefore, we reviewed the randomized controlled studies (RCTs) that were included in Powers et al.’s meta-analysis of PE in 2010 [4] as a starting point and added relevant information from more recent RCTs. We also searched for studies that used data from these RCTs about secondary outcome effects. To that end, we searched in PsychInfo with the following search terms: “prolonged exposure or prolonged exposure therapy,” “PTSD or posttraumatic stress disorder,” and “randomized.” The search was supplemented by consulting experts and reviewing the references in the studies, and inclusion and exclusion criteria were evaluated by all authors. Because we were specifically interested in the secondary effects of PE as a stand-alone therapy, we excluded studies that used PE in combination with any medication or placebo [21–23] or integrated or combined with other psychological treatments [24–27]. As secondary outcomes, we focus on the most frequent comorbid conditions (major depression, anxiety disorders, substance use disorders, personality disorders, and psychotic disorders) and additional symptomatic features (suicidality, dissociation, negative cognitions, negative emotions, and problems with regard to general health and work/social functioning). The main question was the following one: do comorbid symptoms and additional symptomatic features decline along with the PTSD symptoms or at least remain stable as the theoretical models predict? In Table 1, we present an overview of the studies.

**Table 1:** Overview of changes in comorbidity after prolonged exposure for PTSD

	Comorbid disorders					Additional symptomatic features				
	Depression	Anxiety disorders	Substance abuse	Personality disorders	Psychosis	Suicidality	Dissociation	Cognitions	General health	Social/work functioning
RCTs from Powers et al. (2010) meta-analysis and derived secondary outcome papers										
Asukai et al. 2010	CES-D ↓								GHQ28 ↓	Work functioning ↑
Foa et al. 1991	BDI ↓	STAI-S ↓						RAST ↓		
Foa et al. 1999 Cahill et al. 2003	BDI ↓	STAI-S ↓						STAXI-S ↓		SAS global ↑
Foa et al. 2005 Aderka et al. 2013 Foa and Rauch 2004 Moser et al. 2010 Rauch et al. 2009	BDI ↓							PTCI total ↓ PTCI self-blame ↓	PILL frequency ↓	SAS work ↑ SAS social ↑ SAS global ↑
Gilboa et al. 2010	BDI ↓									CGAS ↑
Marks et al. 1998	BDI ↓	STAI-S Not reported FQ Not reported						CAPS guilt ↓ CAPS anger ↓	GHQ not reported	Work/Social Adjustment ↑ Goals ↑
McDonagh et al. 2005	BDI, ns	STAI-S ↓					DES, ns	TSI ↓ CMHS, ns STAXI, ns		QOLI ↑
Nacash et al. 2011	BDI ↓	STAI-S ↓ STAI-T ↓						PTCI ↓		
Power et al. 2002	HADS depression, ns MADRS ↓	HADS anxiety ↓ HAM-A ↓								SDS social ↑ CAPS social ↑
Resick et al. 2002 Galovski et al. 2009 Gradus et al. 2013 Gutner et al. 2013 Resick et al. 2012 Rizvi et al. 2009	BDI ↓ SCID MDD diagnosis ↓					BDI item 9 ↓		TRGI ↓	PSQI ↓ PILL ↓	
Rothbaum et al. 2005	BDI ↓	STAI-S ↓ STAI-T ↓					DES ↓			
Schnurr 2007 Schnurr and Lunney 2012	BDI ↓	STAI-S ↓	ASI alcohol, ns ASI drugs, ns						SF 36 physical, ns	QOLI ↑
Taylor et al. 2003 Stapleton et al. 2006	BDI ↓						CAPS ↓	TRG ↓ TRA ↓ GI-trait ↓ STAXI-trait ↓		
RCTs since 2010 and derived secondary outcome papers										
Shemesh et al. 2011	BDI ↓								Vital signs, cardiac condition, ns	CGI ↑
Pacella et al. 2012 Pacella et al. 2013	CESD ↓		Days of use, ns					PTCI ↓	Physical symptoms ↓ HR-QOL, ns	
Foa et al. 2013	CDI ↓									Global functioning ↑
De Bont et al. 2013					AHRS, ns DRS, ns O-Life ↓	One item, every session, ns				SFS, ns OQ ↓
Van den Berg et al., 2014						One item, every session, ns		PTCI ↓		

↓ Significant pre-post decrease in symptoms, decrease indicates less psychopathology; ↑ significant pre-post increase in functioning, increase indicates better functioning; *ns* no significant pre-post change; *AHRS* Auditory Hallucination Rating Scale; *BDI* Beck Depression Inventory; *CAPS* Clinician Administered PTSD Scale—dissociative items; *CDI* Children’s Depression Inventory; *CES*-*D* Center for Epidemiologic Studies Depression Scale; *CGAS* Children’s Global Assessment Scale; *CGI* Global Clinical Improvement; *CMHS* Cook-Medley Hostility Scale; *DES* Dissociative Experiences Scale; *DRS* Delusion Rating Scale; *FQ* Fear Questionnaire; *GHQ28* General Health Questionnaire, 28 items; *GI* Guilt Inventory Trait; *GPTS* Green Paranoid Thoughts Scale; *HADS* Hospital Anxiety and Depression Scale; *HAM*-*A* Hamilton Anxiety Scale; *HR*-*QOL* Health-Related Quality of Life; *MADRS* Montgomery-Asberg Depression Rating Scale; *O*-*Life* Oxford-Liverpool Inventory of Feelings and Experiences; *OQ* Outcome Questionnaire; *PILL* Pennebaker Inventory of Limbic Languidness; *PSP* Personal and Social Performance Scale; *PSQI* Pittsburgh Sleep Quality Index; *PTCI* Posttraumatic Cognitions Inventory; *QOLI* Quality of Life Inventory; *RAST* Rape Aftermath Symptom Test; *SAS* Social Adjustment Scale; *SCID* Structured Clinical Interview for DSM-IV; *SDS* Sheehan Disability Scale social functioning; *SFS* Social Functioning Scale; *SF36* Short Form Health Survey; *STAI* State (S) and Trait (T) Anxiety Inventory; *STAXI* State-Trait Anger Expression Inventory; *TRA* Trauma-Related Anger—one item; *TRG* Trauma-Related Guilt—one item; *TRGI* Trauma-Related Guilt Inventory; *TSI* Traumatic Stress Institute Beliefs Scale

## Major Depressive Disorder

### Prevalence

Several large epidemiological studies and meta-analysis consistently show an association between PTSD and depression. In a large US epidemiological sample, between 48 and 55 % of individuals with PTSD had comorbid major depressive disorder (MDD) [1]. In another large US epidemiological sample, using tetrachoric correlations, PTSD and MDD were significantly associated with one another (0.50) [28]. Finally, a large meta-analysis (*k* = 57 studies; *N* = 6670) showed that 52 % of individuals with current PTSD had comorbid MDD (95 % CI 48–56 %) [29]. These studies clearly indicate that PTSD and depression often occur together.

### Theoretical Models/Proposed Mechanisms

As mentioned above, PTSD shares diagnostic symptoms with MDD, including anhedonia, difficulty sleeping, irritability, and difficulty concentrating. Besides shared non-specific symptoms, PTSD and MDD may also share underlying constructs such as negative affect, distress, or neuroticism (e.g., [30]). Neuroticism has a substantial genetic loading and may function as a shared vulnerability factor across anxiety and depression (e.g., [31]). Patterns of increased amygdala activation and decreased medial or left dorsolateral prefrontal cortex activation to trauma or negative stimuli are also consistently found in individuals with PTSD and depression (e.g., [32, 33]). There may also be shared genetic, molecular, and neuroanatomical overlap of regulatory mechanisms across fear extinction and depression-like behavior [34]. Finally, shared epigenetic factors may be in play, such that exposure to environmental stress can produce long-lasting changes in DNA methylation related to the pathophysiology of both PTSD and depression (e.g., [35]). These models argue that PTSD and depression symptoms will likely vary together and that successful treatment may have downstream positive effects on epigenic expression.

### Findings From PE RCTs

Of the RCTs for PE, only one [36] reported rates of pre- and post-treatment diagnosis of MDD. At pre-treatment, 47.5 % of those in PE met criteria for MDD; at 9-month follow-up after treatment, 29.5 % of patients in PE had a MDD diagnosis. Of those participating in long-term follow-up (mean 6 years later), only 6.9 % of patients in PE had a MDD diagnosis [37]. Similar, long-term gains were found for depression severity.

All of the major RCTs for PE report depression severity as an outcome measure. When waiting list or non-active control conditions are utilized, PE consistently shows medium to large effect sizes on depression symptoms, which are maintained through follow-up [36, 38–42, 43••, 44, 45, 46••, 47••]. When compared with another trauma-focused treatment, similar size pre- to post- and pre- to follow-up gains are consistently reported [40–42, 48–51]. Thus, there is consistent and strong evidence that PE produces substantial gains in depression, which are maintained over time.

Secondary data analyses from these RCTs help to further isolate how the change process for PTSD and depression occurs. Two studies suggest that changes in PTSD symptoms [52] or related trauma beliefs [53•] drive changes in depression symptoms in PE, rather than the converse. Thus, in PE, there is emerging evidence that reduction in PTSD symptoms promotes reduction in depression symptoms.

### Discussion

As would be expected given the shared symptoms and potential etiological factors, PE shows strong and sustained treatment effects on depression. One of the most striking findings is the consistency of the effects across studies. Further, within PE treatment, changes in PTSD symptoms appear to drive changes in depression, with these findings also replicated in an open trial [54]. Two caveats should be noted. First, RCTs are lacking in military trauma samples, though one large RCT does exist for military sexual assault [45] and open trials are encouraging and show similar effect sizes (e.g., [55, 56]). Second, although the vast majority of the trials allow for comorbid MDD, they typically require that PTSD be the primary diagnosis and that the MDD patient does not have current suicidal intent or plan, though ideation is allowed. Thus, although the reviewed trials routinely include patients with depression in the severe clinical range, the current PE data is limited to patients where trauma exposure and related PTSD symptomatology are more severe than the depression, reflecting appropriately focused clinical care.

## Anxiety Disorders

### Prevalence

Anxiety disorders are among the most common comorbid diagnoses found in individuals with PTSD. In epidemiological research, men and women with PTSD are significantly more likely than those without PTSD to meet criteria for each of the other anxiety disorders (men: OR = 3.0–5.9, women: OR = 2.4–3.4) and prevalence rates for each anxiety disorder range from 7.3 to 31.4 % [1]. Comorbidity rates between PTSD and other anxiety disorders are even higher in clinical samples. For example, a study of civilian outpatients found that those with a primary PTSD diagnosis met criteria for an average of 1.6 comorbid anxiety disorders, with the highest rates found for generalized anxiety disorder (51 %), panic disorder (44 %), and social phobia (43 %) [57].

### Theoretical Models/Proposed Mechanisms

PTSD has been shown to increase the risk of subsequent onset of each of the anxiety disorders, and this elevated risk disappears upon remission of PTSD [58]. This may be due to common underlying mechanisms (e.g., anxiety sensitivity [59]) and/or symptom overlap (e.g., behavioral avoidance of feared situations is common in each anxiety disorder).

### Findings From PE RCTs

Despite the high comorbidity between PTSD and other anxiety disorders, no studies have evaluated the impact of PE on specific anxiety disorder diagnoses or symptoms. However, seven RCTs of PE have included a measure of general anxiety as a secondary outcome [39, 40, 42, 44, 45, 50, 60]. All seven studies reported a significant reduction in general anxiety in PE from pre- to post-treatment, and gains were maintained after treatment at 3 [42], 6 [44, 45, 60], 12 [40], and 15 months [50]. In addition, Power et al. [50] found rates of clinically significant change in anxiety during PE ranging from 29 to 33 % for self-reported anxiety to 62–65 % for interviewer-rated anxiety.

### Discussion

There is strong evidence indicating that PE leads to a significant reduction in general anxiety that is maintained after treatment and no evidence to suggest that PE worsens general anxiety. Although not evaluating PE specifically, a systematic review of PTSD treatment studies involving patients with comorbid panic disorder found that after successful trauma treatment, the majority of patients (56 %) no longer met panic disorder criteria [61]. Of note, the opposite has not been found to be true, namely, successful treatment of panic disorder among individuals with PTSD does not appear to lead to changes in PTSD [62]. Future research would benefit from evaluating the impact of PE on specific comorbid anxiety disorder diagnoses and symptoms.

## Substance Use Disorders

### Prevalence

An association between PTSD, increased substance use, and the development of substance use disorders has been well-documented in the literature [63, 64]. Epidemiological research indicates that approximately two thirds of men and women with PTSD develop a co-occurring substance use disorder, most commonly alcohol, sedative, and cannabis use disorders, either at the same time or subsequent to the onset of PTSD [1, 65].

### Theoretical Models/Proposed Mechanisms

Research examining the functional relationship between PTSD and substance misuse has identified a mediating role of avoidant coping mechanisms (i.e., use of substances to cope, often referred to as self-medication) [66–68], and studies examining the temporal relationship between changes in PTSD and substance use indicate that improvements in PTSD symptom severity are associated with subsequent improvements in substance use [69, 70]. One could therefore assume that substance use outcomes may improve with PE; however, it has only been very recently that studies have examined this relationship.

### Findings From PE RCTs

PE was traditionally considered inappropriate for use among patients with substance use disorders due to safety concerns [71]. These concerns also led to the exclusion of patients with substance dependence (and in some cases abuse) from most trials of PE [8••]. Aside from assessing substance use for the purposes of exclusion, very few studies have included measures of patients’ substance use, and fewer still have reported on them. Of the 13 studies reviewed by Powers et al. [4], only four report having measured patients’ substance use [42, 44, 45, 49] at pre-treatment, and, of those, only one reported on substance use as a secondary outcome [45]. Schnurr and colleagues [45] examined changes in patients’ scores on the Addiction Severity Index (ASI) in relation to alcohol and other drug use (i.e., heroin, methadone, other opiates, barbiturates, sedatives, cocaine, amphetamines, cannabis, hallucinogens, inhalants) as a secondary outcome in their RCT examining the efficacy of PE relative to present-centered therapy (PCT) among 288 female veterans and active duty personnel. At post-treatment and over the follow-up period of 6 months, no significant changes in alcohol or drug use were found for patients who received PE.

Since Powers and colleagues [4] conducted their meta-analysis, Pacella and colleagues [47••] investigated substance use outcomes (defined as the cumulative number of days substances were used in the last month) among 65 patients living with HIV randomized to receive PE or weekly monitoring. Eleven substances were assessed including alcohol, marijuana, cocaine, ecstasy, amphetamines, GHB, PCP, hallucinogens, rohypnol, ketamine, and heroin. Similar to the results of Schnurr and colleagues [45], no significant increases or decreases in substance use were found at post-treatment or at 3-month follow-up.

### Discussion

The small number of studies conducted to date does not provide sufficient evidence to draw firm conclusions regarding the impact of PE on substance use outcomes. Nonetheless, the evidence thus far suggests that PE does not lead to an exacerbation of substance use or severity of substance use disorder. It is recommended that measures of substance use, including tobacco, be included routinely in future research studies and in clinical practice. These assessments should be undertaken pre-, post-, and during treatment to allow for a thorough examination of the relationship between changes in PTSD symptoms and substance use.

## Personality Disorders

### Prevalence

A meta-analysis of 125 studies of clinical and non-clinical samples found that 35 % of adults with PTSD also had at least one personality disorder, with the most common being paranoid (26 %), avoidant (23 %), and borderline (22 %) [72]. Among clinical samples of PTSD patients with various trauma types, the rate of co-occurring personality disorder is even higher (e.g., 39–79 %; [73–75]).

### Theoretical Model

Several theories have been proposed to account for the high comorbidity between PTSD and personality disorders. Childhood abuse and adversity have been implicated in the development of both PTSD and personality disorders (e.g., [13, 76, 77]). In addition, PTSD may maintain or exacerbate problems associated with personality disorders. For example, PTSD has been found to increase core features of borderline personality disorder such as emotion dysregulation and recurrent suicidal and self-injurious behavior (e.g., [25]). Finally, the high comorbidity may be at least partially explained by symptom overlap between PTSD and some personality disorders, such as affective reactivity in borderline personality disorder and detachment from others in paranoid personality disorder. These models suggest that improvements in PTSD would likely lead to improvements in personality disorders that either developed in response to traumatic events or are being exacerbated by PTSD and associated problems.

### Findings From PE RCTs

None of the RCTs of PE in this review excluded individuals on the basis of a personality disorder diagnosis. However, individuals with a primary diagnosis of a personality disorder would have been excluded from most studies. Although several studies have evaluated the impact of personality disorders on PTSD and related symptoms after PE [75, 78, 79], to date, no studies have yet evaluated the impact of PE on personality disorder symptoms.

### Discussion

Given the high prevalence of personality disorders among individuals with PTSD, the ability of PE to reduce co-occurring personality disorder symptoms is an important question to examine in future research.

## Psychosis

### Prevalence

The rate of PTSD in patients with a psychotic disorder is estimated to be 12.4 % (95 % CI 4.0–20.8 %; [80]). Despite this relatively high rate of comorbidity, in clinical practice, PTSD is largely underdiagnosed in this population. Conversely, psychotic symptoms occur frequently in PTSD patients (15–64 %; see for a review [81]), although others find lower rates (17 %) when other comorbid conditions are controlled [82].

### Theoretical Models/Proposed Mechanisms

There is, however, much attention in regard to the relationship among trauma, PTSD, and psychosis [83–86]. Many studies found evidence for the causal relationship between childhood trauma and the development of psychosis [87]. Further, in many models, it is stressed that symptoms of PTSD and psychosis negatively interact with each other [10, 84], that they share some symptoms (such as intrusions and paranoia), and that they share developmental and maintenance processes (e.g., a self-blaming attributional style and avoidance behavior) [84]. Based on these models, it can be understood that when PTSD symptoms decrease during treatment, the psychotic symptoms may decrease along with them.

### Findings From PE RCTs

Despite these strong relationships between PTSD and psychosis, little is known about the effects of trauma-focused treatment such as PE in this patient population. Most clinicians fear that this patient population may be too vulnerable to tolerate trauma-focused treatments [6], and psychosis (especially not medically stabilized and current psychosis) is a frequently used exclusion criterion in PTSD treatment trials [88]. Accordingly, in most studies of Powers’ meta-analysis [4], patients with a current and/or past psychosis were excluded; consequently, these studies do not provide information about changes in psychotic symptoms during PE.

More recently, several studies have been conducted that include patients with psychotic disorders, including current psychotic disorders and schizophrenia. In a multiple baseline randomized controlled design, de Bont and colleagues [89••] found evidence for the feasibility, effectiveness, and safety of trauma-focussed treatments, including PE, in either past or present psychotic patients (*N* = 10), with comparable effect sizes as in PTSD populations without psychosis. In addition, no treatment-related increases were found in hallucinations or delusions during or after PE. In addition, psychosis-prone thinking significantly declined during treatment. In a large multi-site RCT with PTSD patients with past or present psychotic disorder (the majority of patients had schizophrenia or schizoaffective disorder; *N* = 155 [90••]), PE was found to be feasible, safe, and effective, with large effect sizes that are comparable to studies including PTSD patients without psychosis. Compared with waiting list, no more severe adverse events (including psychiatric hospital admissions and suicide attempts) occurred during PE.

### Discussion

In conclusion, though many more studies are necessary, the available research indicates that some psychotic symptoms (especially psychosis-prone thinking) decline along with PTSD symptoms after PE. The changes in psychosis-related thinking may be related to changes in trauma-related cognitions, such as beliefs about safety and trust, as these cognitions have a substantial overlap with delusional and paranoid thinking. No changes were found for other psychotic symptoms (e.g., hallucinations). Notably, no increases in psychotic symptoms were found during PE as many clinicians fear.

## Suicidality

### Prevalence

Individuals with PTSD are at heightened risk of suicidal ideation and behavior. A recent meta-analysis found a large overall effect size (*g* = 0.78) for the association between PTSD and suicidality, and this link was significant for both suicidal ideation and suicide attempts, across different trauma samples (e.g., combat veterans, physical/sexual abuse, natural disasters) and in clinical and non-clinical samples [91].

### Theoretical Models/Proposed Mechanisms

Several theoretical models have been proposed to explain the relationship between PTSD and suicidality. Some models suggest that certain PTSD symptoms directly increase suicide risk; for example, more severe re-experiencing and numbing symptoms, including physiological reactivity to trauma cues, inability to recall parts of the trauma, and a sense of a foreshortened future are directly associated with suicide attempts [92]. Other models highlight the role of various cognitive-affective processes as direct or indirect mediators between PTSD and suicidality, including perceptions of defeat and entrapment (e.g., [93]), negative self-appraisals (e.g., [94]), hopelessness (e.g., [91]), and guilt and shame (e.g., [95]). In addition, some models propose that the link between PTSD and suicidal behavior is partially explained and/or compounded by other comorbid problems such as functional impairment [96], depression [97], sleep disturbance [98], and alcohol dependence [99]. Taken together, these models suggest that treating PTSD is likely to reduce suicidality directly by reducing PTSD symptoms and indirectly by reducing established cognitive and affective mediators as well as other compounding problems.

### Findings From PE RCTs

Despite the high rate of suicidality among individuals with PTSD, suicide-related outcomes are rarely reported in PTSD treatment research, including studies of PE. This is likely due to the fact that acutely suicidal patients are typically excluded from these studies [88]. The exclusion of acutely suicidal patients is due to concerns that trauma-focused treatment may exacerbate suicidality (e.g., [6]). As with other PTSD treatments, PE excludes individuals with a recent history of suicidal or severe self-injurious behavior (typically the last 3 months) and/or individuals believed to be at imminent risk of suicide such as those with suicidal intent and/or a plan [100]. Although patients with non-acute suicidal ideation are generally included in PE treatment studies, only one study has evaluated the impact of PE on suicidal ideation [101]. This study used secondary data from the RCT [36] of female rape survivors with chronic PTSD in which participants were excluded if they reported suicidal intent or current parasuicidal behavior. In this study, the proportion of PE patients endorsing any suicidal ideation on a single-item measure decreased significantly during treatment (from approximately 24 to 10 %) with further slight decreases found across up to 10 years of follow-up [101]. However, changes in PTSD did not predict reductions in suicidal ideation in PE after controlling for the effects of major depression and hopelessness. Thus, the mechanisms underlying the reduction of suicidal ideation in PE were not clear.

In addition, one study evaluated the rate of suicidal behavior and non-suicidal self-injury during PE among five patients with comorbid PTSD and psychotic disorders [89••]. This study excluded patients with acute suicidality but included those with non-acute suicidal ideation. Suicidal and non-suicidal self-injury were measured at every treatment session, and no instances of either behavior were reported during PE, indicating that suicidality does not increase during PE. In a large randomized controlled trial of 155 patients with comorbid PTSD and psychotic disorders [90••], patients with higher levels of suicidality were included. In PE, at baseline, 62.3 % had a history of attempting suicide and 50.9 % had current medium or high levels of suicide risk. Suicidality was checked every session, and in comparison with the other treatments (active treatment and waiting list), no more adverse events involving suicidality occurred during PE.

### Discussion

There is no evidence that PE exacerbates suicidality, and there are no reports of completed suicides in any PE study. However, there is currently insufficient research to draw firm conclusions about the impact of PE on suicide-related outcomes. Although the available research does not support common clinician concerns that PE may exacerbate suicidality, it is also limited by its exclusion of individuals with acute suicidality and its reliance on relatively low-risk samples with low base rates of suicidality. Future research would benefit from the inclusion of standardized measures of suicidality both during and after PE. In addition, studies are needed to evaluate whether these effects generalize to higher risk populations, such as those with a recent history of suicidal behavior and/or current suicidal intent or plans, or whether treatments that include a stabilization phase prior to trauma processing may be necessary for higher risk patients (e.g., [102]).

## Dissociation

### Prevalence

Many PTSD patients have at least some symptoms of dissociation. Some dissociative symptoms, such as flashbacks, numbing, and psychogenic amnesia, are included in the PTSD DSM-5 diagnostic criteria and are, as such, considered part of the diagnosis of PTSD. Other dissociative symptoms, such as derealization, depersonalization, and current reduction in awareness are usually established apart from the PTSD diagnosis. For patients scoring high on these symptoms, the DSM-5 [103] has a “dissociative subtype” specifier. In a veteran sample, 12 % of the PTSD patients showed elevated symptoms of derealization and depersonalization [104]. Other studies showed that 15 % (men, Vietnam veterans) to 30 % (women, veterans with high rates of sexual abuse) [105] of PTSD patients were classified into the dissociative subtype. In civilian samples, a 15–25 % rate of this dissociative subtype was found [106, 107]. In sum, about 12–30 % of the PTSD patients show elevated dissociative symptoms. However, it should be noted that other studies did not find dissociation to be a taxon in PTSD but instead situated on a dimensional scale within PTSD [108, 109], suggesting that as PTSD symptoms increase, so do dissociative symptoms and vice versa.

### Theoretical Models/Proposed Mechanisms

Many clinicians (51 %) assume that *any form of dissociation* is a contraindication for exposure therapy [6]. Theoretically, it is assumed that severe dissociation may hinder fear activation and thereby emotional processing, which is a crucial element underlying the working mechanism of PE [110]. In line with this view (see for an overview [111]), dissociation is regarded as a coping strategy to deal with the extreme arousal elicited by recalling of trauma-related memories during exposure. This coping strategy is referred to as “emotional overmodulation” which leads to hyperinhibition of the amygdala activity, with fear inhibition as a result. Therefore, it is assumed that the emotional arousal that is evoked by PE may lead to an increase in dissociation in order to cope with the high fear levels. However, one could also reason that when PTSD symptoms decline during treatment, this dissociative coping strategy is less needed, and as a result, dissociative symptoms will also decrease.

### Findings From PE RCTs

In three of the studies included in the meta-analysis of Powers, dissociation was measured as a secondary outcome measure. In a sample of childhood sexual abuse victims, after exposure therapy, self-reported symptoms of dissociation (Dissociative Experiences Scale [DES] [112, 113]) decreased, though this decline was not significant [60]. In rape victims, self-reported symptoms of dissociation (DES) decreased significantly after PE [44]. In victims of mixed trauma, symptoms of numbing (assessed via the Clinician-Administered PTSD Scale [CAPS] numbing subscale) significantly declined after PE [51]. At post-treatment, 47 % of the patients that received PE showed clinically significant reductions in numbing; at 3 months follow-up, this was 53 %. In addition, clinician-rated dissociative symptoms, as measured with three additional items of the CAPS, significantly declined after PE.

### Discussion

Although dissociative symptoms are thus far not systematically evaluated after PE, the studies that did include dissociation showed that dissociative symptoms, both self-reported and clinician-rated, significantly decline after PE. It is, however, not known if these decreases in dissociative symptoms also hold for PTSD patients with severe dissociative symptoms, for instance patients that fulfill diagnostic criteria of the PTSD dissociative subtype. In light of the newly introduced diagnostic dissociative subtype of PTSD, it is strongly recommended that future studies include secondary measures (both self-report and clinician-rated) of derealization and depersonalization. Further, dissociation is thus far only operationalized in terms of detachment symptoms, while dissociation is a broader concept (see [114]). Therefore, it is recommended that also somatoform dissociation and symptoms of compartmentalization, such as conversion disorder symptoms or symptoms of tonic immobility, are included.

## Negative Cognitions and Emotions

### Prevalence

Negative cognitions about oneself (e.g., I am incompetent), the world (e.g., No one can be trusted), and self-blame (e.g., I should have been able to stop it), as well as negative emotions such as shame, guilt, and anger, are commonly associated with PTSD, though not every individual with PTSD reports these at extreme or dysfunctional levels. In a recent US national epidemiological study, of those with probable PTSD, 35 % reported negative beliefs, 34 % guilt or shame, and 30 % anger or aggression symptoms in the past month [115]. These numbers are in contrast with other PTSD symptoms which occur at higher levels among those with probable PTSD, such as re-experiencing (52 %) and avoidance of trauma-related thoughts (53 %; [115]). This is consistent with these secondary symptoms being much more related to idiosyncratic trauma characteristics (e.g., I did not stop my sister from also being abused) or pre-existing individual differences (e.g., I always had a temper).

### Proposed Working Mechanism

Although it may not be intuitive to some that an exposure-based treatment such as PE would target these secondary symptoms, particularly without elements such as cognitive restructuring or anger management, current theories argue against this. Emotional processing theory emphasizes the role of providing corrective information and altering cognitive meaning elements of the fear schema as critical for recovery; in contrast, anger is viewed as a form of avoidance that impedes accessing the fear schema and recovery [5]. Other theorists also highlight the role of new inhibitory learning (e.g., [116]), cognitive shifts during exposure (e.g., [117]), and changes in negative appraisals [118] underlying recovery.

Recently, Litz and colleagues [119] put forth the construct of moral injury, arguing that particularly with combat trauma, individuals may act in ways that contradict deeply held moral beliefs or experience conflict about the unethical behaviors of others. They suggest interventions such as PE may not be sufficient to address these issues. Others have argued that PE is able to flexibly address guilt, shame, and anger related to moral injury (e.g., [120]). Accordingly, although some theorists have raised concerns, main theoretical models predict that these secondary symptoms will reduce as PTSD symptoms reduce or may even underlie reductions in PTSD symptoms.

### Results From PE RCTs

Secondary data analyses, reporting psychometrically validated measures of these constructs in the large randomized trials, show small to large decreases and sustained improvement in negative cognitions [39, 42, 47••, 60, 90••, 121], trauma-related guilt [36, 122], and anger [122, 123]. One study [60] failed to find changes in hostility and anger, though in this study improvement in other domains including PTSD was also limited arguing against this finding being specific in regard to anger. Single-item measures of trauma-related guilt and anger also show moderate to large pre- to post-treatment and pre- to follow-up effects for PE [49, 51]. Though some have raised concerns that initial anger may impede PTSD symptom reduction, increase PE dropout, or worsen these symptoms, this has not been consistently seen [122, 123]. Variability in the expression of these associated features may help explain the range of observed effects; that is, an individual with little or no initial anger, guilt, or distorted cognitions has no statistical room for improvement. Indeed, effect sizes actually tend to increase (e.g., [123]) or stay the same [122] when individuals with more extreme initial scores are examined.

### Discussion

Taken together, these studies argue that PE reduces negative cognitions and emotions without explicit cognitive restructuring procedures. Across studies, particularly for those with more severe negative beliefs, guilt or shame, and anger, PE reduces these secondary symptoms. In particular, for guilt, shame, and anger, the routine use of psychometrically validated measures is needed to further strengthen these conclusions. Changes in cognitions are related to changes in PTSD severity [121] or may even drive these changes [53•]. Interestingly, the explicit addition of cognitive restructuring does not appear to augment cognitive changes [121] and may even impair changes for those with extreme negative cognitions [124]. Additional research with larger samples and subsamples of males and veterans is needed.

## General Health and Work/Social Functioning

### Prevalence

PTSD is related to both general health problems and lower social functioning. In a meta-analysis, Pacella and colleagues [47••] found elevated rates of general physical health problems and general medical conditions in persons with PTSD. In addition, specific medical conditions were found to be related to PTSD, such as cardiovascular symptoms, gastrointestinal symptoms, and pain.

Social functioning is generally low among PTSD patients; for instance, 70 % of PTSD veterans and their partners reported clinically significant levels of relational distress [125]. Work and school functioning is also lowered and social contacts are limited. In line, in a sample of primary care anxiety patients, of all anxiety disorders, PTSD was most consistently related to lower levels of functioning, including general health and social functioning [126].

### Rationale/Proposed Working Mechanism

Several studies showed that PTSD symptoms mediated the relationship between traumatic experiences and general health problems (see for a review [127]). Several studies have shown that PTSD-related biological markers such as abnormal electrocardiograph (ECG) results, high white blood cell counts, and T cell counts [128] can explain high rates of cardiovascular and auto-immune diseases among PTSD patients.

In a meta-analysis [129], lack of social support was strongly related to the development of PTSD, indicating that good social functioning contacts may be a buffer against post-traumatic stress. Conversely, however, specific PTSD symptoms, such as numbing, lack of interest, and cognitive distortions about safety and trust, may cause problems in social functioning. Accordingly, in a large sample (*N* = 2249), it was indeed found that PTSD predicted lack of social support [130].

Because general health problems and lower social functioning after trauma have been shown to be moderated by PTSD symptoms, it is expected that general health and social functioning will increase in line with a decrease in PTSD symptoms.

### Results From PE RCTs

In most studies that included health as an outcome measure, general (physical) health problems were found to be significantly improved after PE [38, 131, 132], as was sleep quality [131, 133]. Also, in a more recent RCT in a medical sample (HIV patients), evidence was found for improvements of physical health and increased short-term adherence to medical medication after PE [47••]. What is more, in the Rauch et al. study [47••], evidence was found that changes in PTSD symptoms (and not in depressive symptoms) contributed to the change in physical health. For patients with PTSD due to a cardiovascular event, it was shown that there were no significant relevant increases in vital signs during or after PE, including blood pressure, arterial pressure, and pulse [46••]. Also, no adverse events, such as death or recurrent myocardial infarctions, happened.

In most studies, work and social functioning were included as secondary outcome measures. In all studies that measured work and social functioning, significant increases were found in work functioning [38, 41, 49] and quality of life [45, 60] after PE. Also, social adjustment and functioning [40, 41, 43••, 49, 50] increased after PE. In addition, quality of life was significantly improved after PE [45, 60].

### Discussion

In line with the hypotheses, based on the idea that PTSD symptoms moderate the relationship between trauma and general functioning, the findings were very consistent in that both general health and social functioning improved along with the PTSD symptoms after PE. This is highly important information in light of cost-effectiveness issues, given the fact that PTSD patients have high medical care consumption and low social and work functioning. In terms of physical vital signs, PE proved to be not dangerous, not even in a vulnerable patient group with cardiovascular problems. That finding suggests that PE can be safely provided for medical populations also.

In only a few studies, general health was included as a secondary measure. Given the high medical consumption of PTSD patients, however, and especially for cost-effectiveness purposes, it may be recommended to include this measure in future studies. The same goes for social functioning and work functioning. Additionally, given that social support is an important predictor of PTSD symptoms, changes in social support, as a specific element of social functioning, may be crucial to include as a secondary measure, for instance to predict relapse in PTSD symptoms.

## Conclusions

In this review, we presented an overview of results regarding secondary outcomes of PE, including several comorbid conditions and additional symptomatic features. For some areas of comorbidity, there is strong evidence that PE leads to improvements in these secondary outcomes: depression, general anxiety, trauma-related cognitions, and overall functioning. For other comorbid symptoms, however, relatively little systematic research has been done, not all relevant comorbid conditions were included, and secondary outcome measures varied highly across the studies, thereby limiting the ability to draw firm conclusions. That being said, based on the available data, a very consistent picture emerged from all studies and across all secondary outcomes. Consistent with our hypothesis, results consistently showed that comorbid conditions and additional symptomatic features decreased along with the PTSD symptoms (depression, general anxiety, problems in social and work functioning, dissociation, physical health problems, trauma-related negative cognitions and emotions) or at least did not increase during PE (substance abuse, delusions, hallucinations, suicidality). None of the studies found an increase for any of the comorbid conditions under study.

These findings are in line with several theoretical models about PTSD and comorbidity, as outlined in the “Introduction” section. In these models, PTSD symptoms are usually regarded as moderators and/or mediators of the relationship between trauma and comorbid conditions, suggesting that when PTSD symptoms decrease, the associated comorbid conditions are likely to also decrease. Of note, however, few studies have evaluated whether changes in PTSD are directly or indirectly associated with improvements in secondary outcomes. For example, PE has been shown to significantly reduce depression in all studies, and it is possible that changes in some comorbid conditions (e.g., suicidality, social functioning) after PE may be specifically attributed to a decline in depression instead of or in addition to a decline in PTSD symptoms. This may be an indirect process when, for example, PTSD symptoms change first and lead to subsequent changes in depression [52]. Alternatively, reductions in PTSD symptoms, but not depression, may be directly associated with improvements in other areas; for example, changes in physical health after PE are more related to changes in PTSD symptoms than to changes in depression [134]. In future studies, to shed more light on the underlying mechanisms, it is recommended that direct and indirect pathways between changes in PTSD and secondary outcomes are evaluated.

Some comorbid conditions and associated symptoms were more likely to change as a result of a decrease of PTSD symptoms than others. Depression and general anxiety, for instance, were consistently found to decrease along with the PTSD symptoms, while other conditions, such as substance abuse and hallucinations, tended to remain stable. This may be due to the fact that some disorders (e.g., major depression and PTSD) share more symptomatology and underlying etiology than others (e.g., psychotic disorders and PTSD). Also, comorbid disorders may improve in some patients, and not in others, due to different pathways for the comorbidity (e.g., [18]). For example, a patient could first have an onset of psychosis and then be exposed to trauma and develop PTSD. Or a patient could have experienced childhood sexual assault, developed PTSD, and then, when he or she reached a high-risk age, developed psychosis. This differential relatedness of comorbid conditions with PTSD symptoms may lead to differences in changes of comorbid symptoms a result of PTSD treatment. Therefore, it is recommended that future studies explicitly attempt to assess the interrelatedness, including timing of onset, between the comorbid conditions and PTSD.

These findings have several implications. From a clinical point of view, these studies consistently show that fears of long-term exacerbation of comorbid conditions or deterioration of functioning as a result of PE are not valid reasons for excluding many PTSD patients from an effective trauma-focused treatment. It was not our goal in this article to evaluate the efficacy of PE with regard to decreases in comorbid symptoms in comparison to other trauma-focused treatments, such as cognitive therapy or eye movement desensitization and reprocessing (EMDR). Based on the theoretical models, changes in comorbid symptoms are not expected to relate exclusively to PE but may generalize to other effective trauma-focused treatments as well. Note, however, that for other trauma-focused treatments, different pathways to changes in comorbid conditions may appear. For PE, the conclusion that comorbid symptoms do not increase by the end of treatment is especially of importance given that some clinicians are usually more hesitant to use PE in patients with comorbid conditions than other trauma-focused treatments like EMDR [7].

We deliberately chose to include only studies in our review in which PE was used as a stand-alone therapy and excluded studies that used additional treatment elements such as emotion regulation skills, cognitive therapy, pharmacological agents, and studies that used integrated treatment approaches in which both PTSD and the comorbid symptoms are targeted at the same time. Therefore, we were able to review changes in comorbid conditions after PE without the comorbid conditions being explicitly targeted. Our findings that many comorbid conditions significantly improve after PE, perhaps as a result of improvement in PTSD symptoms, call into question the need to add interventions aimed to change comorbid conditions to PE. Instead, it may be a better approach to evaluate the remaining comorbid symptoms and associated features after PE and, if needed, provide targeted interventions to achieve full remission for these symptoms.

These findings also question the necessity of phase-oriented treatment approaches in cases of PTSD and comorbid conditions (see [24]). In these treatments, the first phase is usually directly aimed at coping skills to deal with or decrease comorbid conditions, such as emotion regulation problems (including dissociation) and poor interpersonal skills. In the second phase, PTSD symptoms are directly targeted via trauma-focused treatments such as PE. The rationale for a phase-oriented treatment approach is that these comorbid symptoms, when left untreated, will interfere with trauma-focused treatment, or will increase when the trauma is directly processed, and must therefore be addressed before trauma processing. In an RCT, a phase-oriented approach such as STAIR-PE was found to be more effective than support plus PE [24], but studies that directly compare a phase-oriented approach and PE without any pre-phase are still lacking. In our review, however, we found no evidence for this phase-oriented view. Instead, we found that when PTSD symptoms were directly targeted without a preparatory treatment phase, comorbid symptoms decreased along with the PTSD symptoms or at least remained stable. Moreover, a recent study found evidence for improvements in emotion regulation after PE as a stand-alone therapy [135•].

Our findings are consistent with theoretical models proposing that several comorbid conditions may derive directly or indirectly from the PTSD symptoms [10, 18]. Therefore, directly targeting the PTSD symptoms with a trauma-focused treatment such as PE would be a logical first step in treatment. In contrast, adjunctive treatments targeted at specific secondary conditions are typically longer and may unnecessarily postpone decreases in PTSD symptoms and related comorbidity or even increase the risk of dropout before the patient arrives at the trauma-focused treatment phase. For comorbid conditions that do not seem to change during PE, such as substance abuse, integrated treatments in which the PTSD symptoms and the comorbidity are targeted at the same time are recommended (see [26]). In addition, for comorbid conditions such as acute suicidality for which PE has not yet been evaluated as a stand-alone treatment, it is recommended to use an integrated treatment that focuses on stabilizing suicidality prior to starting trauma-focused treatment and continues to monitor and target suicidality as needed during PE (e.g., [102]). Future research should be aimed at directly comparing phase-oriented treatments to PE-alone or integrated treatments. Also, a reverse phase-oriented treatment may be considered in which PTSD symptoms are targeted in the first phase and remaining comorbid symptoms are addressed in the second phase.

From a cost-effectiveness point of view, the finding that both PTSD and secondary outcomes tend to systematically decrease may mean that those patients need less care following their PE treatment. This was nicely illustrated by a study of Tuerk et al. [136] that measured the mental health service use of veterans with PTSD in the year before and the year after PE. They found that for patients who completed PE, the use of health services decreased significantly. Le and colleagues [137] also reported the cost-effectiveness of PE in the year following treatment, accounting for the cost of usage of both mental health and broader health services.

One limitation of our review is that the outcome measures were evaluated at the end of treatment, and clinicians may worry about exacerbations of PTSD symptoms and related comorbidity during treatment, especially in the weeks after the initiation of imaginal exposure. Indeed, there are indications that the process of PTSD symptom reduction during PE is quadratic, as symptoms, such as re-experiencing symptoms, may worsen in the first weeks of treatment before they get better [138]. As a result, because PTSD symptoms may be functionally related to some comorbid conditions, the comorbid symptoms may also temporarily increase after initiation of imaginal exposure. Foa and colleagues [139] specifically studied these patterns of exacerbation of symptoms during PE and found that only a minority of patients showed exacerbations of PTSD symptoms (15.4 %) or comorbid symptoms (general anxiety 28.2 %; depression 12.8 %) after the first imaginal exposure session. Importantly, however, these exacerbations were temporary and unrelated to treatment outcome and dropout. This means that when a clinician is faced with increases in symptoms (either PTSD or comorbid symptoms) after initiation of imaginal exposure, it can be expected that if PE is continued, the symptoms will eventually decrease significantly and the patient will profit as much from the treatment as patients without initial exacerbations. This is further reified by a recent large study (*N* = 361) of pooled data across RCTs [140] showing that, at post-treatment, reliable worsening of PTSD symptoms after PE was non-existent and reliable worsening of depression was low (1.5 %), with no differences from other trauma-focused treatments (CPT, EMDR).

Another limitation is that comorbidity measures that were included in the studies were usually measured with self-reported severity or frequency of symptoms. Therefore, its clinical value may be limited because, despite a statistical decrease in symptoms, it remains unknown if patients still fulfill diagnostic criteria for the specific comorbid disorder and/or whether this decrease is clinically significant. It is therefore recommended that future studies evaluate both the clinical and statistical significance of reductions in comorbid conditions.

For some comorbid conditions, such as personality disorders, secondary outcome measures were lacking in the literature. Some of this may be an inherent assumption (which may not be accurate) that personality traits or disorders are fixed and stable and would be unlikely to change with any form of treatment. This lack of measurement was also the case for some comorbid conditions that may be of importance, especially for some specific patient populations. For instance, for studies addressing treatment outcomes in patients with “complex” PTSD, it may be of importance to include measures about emotion regulation, self-esteem, and interpersonal relationships, given their interrelationship with PTSD. Consistent with our findings, a recent study indicated that problems with emotion regulation also diminished after PE [135•] in patients with a history of childhood abuse, without any emotion regulation skill training.

In conclusion, the impact of trauma-focused treatments such as PE on comorbid conditions is highly clinically relevant given that a vast majority of PTSD patients present with comorbid problems. Although systematic research is not available for all comorbid disorders, in the available studies, a very consistent pattern was found, namely, comorbid disorders and related symptoms tend to decline along with PTSD symptoms or at least did not increase as a result of PE. This is consistent with theoretical models emphasizing the moderating and mediating role of PTSD symptoms between trauma and psychopathology [10, 18]. Clinically, a fear of exacerbation of comorbid symptoms as a result of PE—given the populations that have currently been studied—seems to not be a valid reason for exclusion of patients from PE.
